# Outcomes of Pregnancy and Recurrence of Pelvic Organ Prolapse After Laparoscopic Sacrocolpopexy With Uterine Preservation: A Retrospective Case-Series Study

**DOI:** 10.7759/cureus.37874

**Published:** 2023-04-20

**Authors:** Saeed Alsary, Jawaher Alsahabi, Maha Al Baalharith

**Affiliations:** 1 Department of Obstetrics and Gynecology, Urogynecology Division, Ministry of the National Guard - Health Affairs, Riyadh, SAU; 2 Department of Obstetrics and Gynecology, King Saud Bin Abdulaziz University for Health Sciences, Riyadh, SAU

**Keywords:** uterus, prolene mesh, sacrocolpopexy (scp), pregnancy, pelvic organ prolapse (pop), laparoscopy

## Abstract

Introduction: The objective is to study the pregnancy outcomes and the recurrence of pelvic organ prolapse (POP) following laparoscopic sacrocolpopexy (SCP) with uterine preservation in patients who are considering future fertility.

Methods: This is a retrospective study in single teaching hospital. The study included six young women who conceived spontaneously, after laparoscopic SCP. Data were retrieved from the medical records of patients who underwent laparoscopic SCP and became pregnant after surgery.

Results: A total of six women conceived spontaneously. Two of them got pregnant twice. The total number of pregnancies was considered to be eight. Two pregnancies ended in spontaneous miscarriages; one of them required dilatation and curettage. Five pregnancies were carried out to term, and one pregnancy ended by preterm delivery at 32 weeks. All the neonates were at appropriate weight as per their respective gestational ages except the one preterm delivery, which was small for gestational age. No intraoperative difficulties were reported during all cesarean sections. Follow-up was documented by objective assessment for more than four years post laparoscopic SCP. No recurrent apical prolapse was found. Only one patient had a recurrent, symptomatic, grade two cystocele that required reoperation.

Conclusion: Patients who desired fertility and presented with symptomatic high-grade POP were good candidates for laparoscopic SCP. Our findings demonstrate the visibility of laparoscopic SCP as an effective surgical intervention that not only levitates symptomatic POP but preserves fertility in young women.

## Introduction

Pelvic organ prolapse (POP) is a benign disorder that causes bothersome functional symptoms. Millions of women throughout the world suffer from POP. Up to 30% of the population may be affected by POP, according to Olsen et al. [1]. In young females with high-grade POP, preserving fertility is of paramount importance. The pelvic organs are subjected to strain during pregnancy and childbirth, especially in those women who currently suffer from a prolapse or are predisposed to acquiring one. Traditionally, the main curative procedure for high-grade POP in women is a hysterectomy. Nevertheless, Pandeva et al. noted that hysterectomy alone does not treat prolapse, which typically results from a lack of support from the cardinal and uterosacral ligaments [2]. The minimally invasive laparoscopic sacrocolpopexy (SCP) maintains the benefits of the abdominal SCP as the gold standard surgery for POP [3]. In terms of faster operating times, fewer intraoperative complications, and mesh erosions, Constantini et al. and Meriwether et al. indicated that laparoscopic SCP with uterine preservation is preferred to SCP with hysterectomy for the management of POP [4,5]. Although SCP is a common surgical procedure for high-grade POP, there is inadequate clinical data to support its durability throughout and after pregnancy and neonatal outcomes. The benefit of laparoscopic SCP without hysterectomy in maintaining fertility and treating POP was underlined by this study. Afterward, successful pregnancy and delivery were achieved. Such a procedure was not associated with the recurrence of apical prolapse in all cases. As compared to earlier studies that have been published, our retrospective case series has the longest follow-up period.

## Materials and methods

The regulations of King Abdullah International Medical Research Center (KAIMRC), Ministry of National Guard - Health Affairs, were followed, and the study was approved by the Institutional Review Board of KAIMRC, Riyadh, Saudi Arabia. Data were retrieved from the medical records of young females who underwent laparoscopic SCP with uterine preservation between July 2009 and December 2019 at King Abdul Aziz Medical City, Ministry of National Guard - Health Affairs, Riyadh, Saudi Arabia. There were 36 patients identified during this period. Six of these patients spontaneously became pregnant, and all six had cesarean deliveries.

Patients involved in this case series underwent staging of POP by a senior urogynecologist using POP quantification (POP-Q) scoring system. All patients included in this case series were called for clinical assessment by our team prior to publication. All patients had pelvic ultrasounds prior to surgery. Patients with stress urinary incontinence were not included in the urodynamic investigation if there was no clinical suspension of voiding dysfunction. All patients either had failed conservative measures such as vaginal pessary or declined such measures and asked to go for corrective surgery from the beginning.

Surgical technique

**Figure 1:**
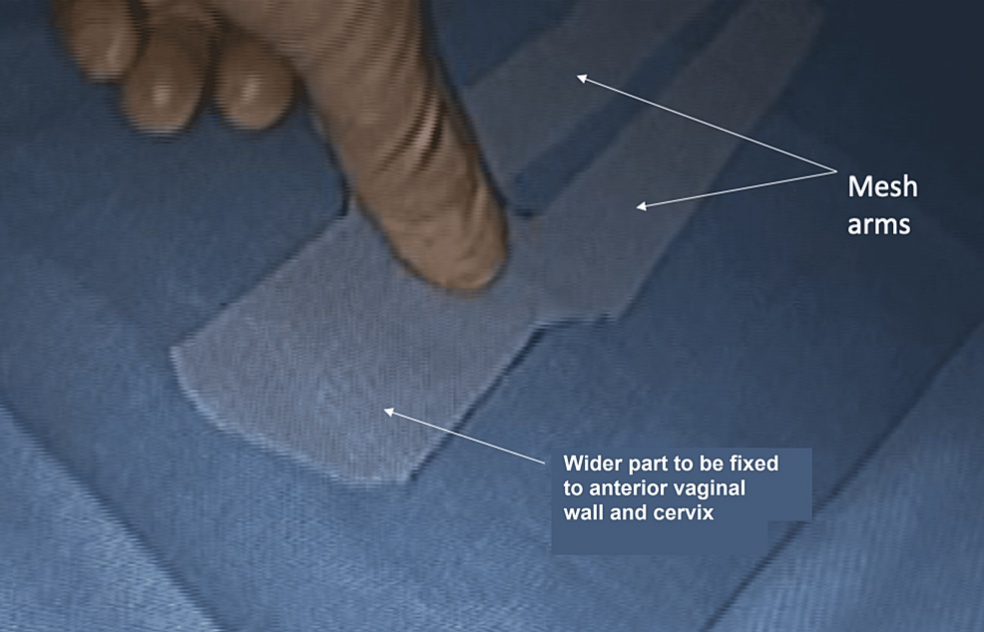
Mesh type and tailing: monofilament polypropylene mesh is used.

Laparoscopic SCP is a mesh-based procedure. Our surgical technique requires using a single (anterior) monofilament polypropylene mesh. The mesh is shaped to look like a tuning fork (Figure 1).

**Figure 2:**
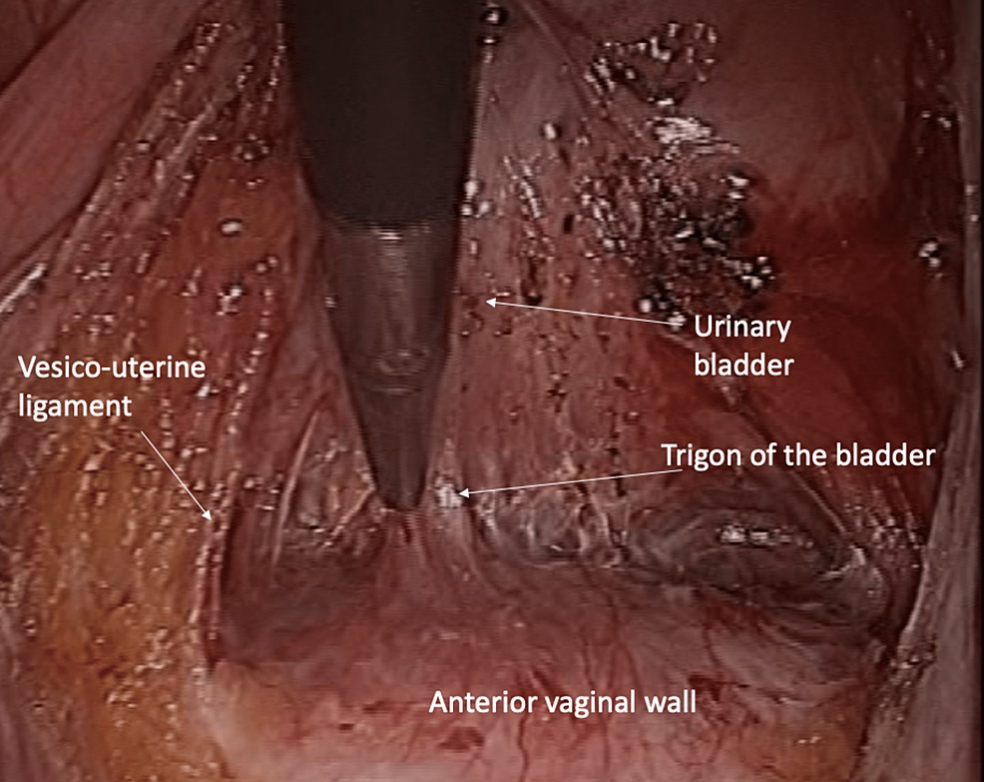
Anterior dissection to develop the vesicovaginal space.

The procedure starts by opening the peritoneum above the sacral promontory with careful exposure of the underlining longitudinal intervertebral ligaments. Further opening of the peritoneum is followed to arrive at the pouch of Douglas between the right ureter and the lateral border of sigmoid colon. Decent peritoneal mobilization is needed to cover the mesh later on without causing tension on the adjacent organs like sigmoid colon or right ureter. Fenestrations are then made at the broad ligament above the level of the uterine artery on each side. The anterior dissection is done to develop the vesicovaginal space (Figure 2) and to expose the anterior vaginal wall.

**Figure 3:**
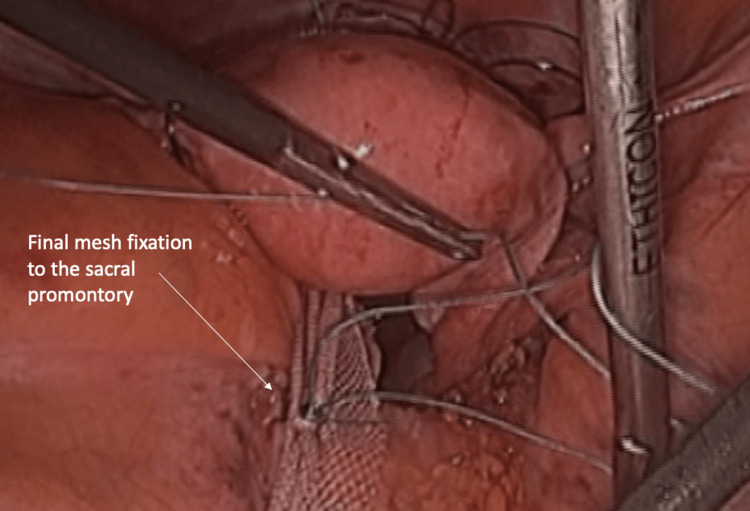
Final mesh fixation to the sacral promontory.

The wider part of the mesh is then sutured to the anterior vaginal wall and cervix using nonabsorbable ETHIBOND stitches (Ethicon, Inc., Raritan, New Jersey, United States). Five 3-0 interrupted stitches are used to fix the mesh to the anterior vaginal wall, and two 2-0 stitches are used at the cervix level, all using extracorporeal notes. The mesh arms are then passed through the created fenestrations in the broad ligaments of the uterus. These two mesh arms are finally retrieved posteriorly and fixed to the sacral promontory (Figure 3) using zero nonabsorbable ETHIBOND suture.

Proper tension on the mesh is made to correct the anterior and apical prolapse. It is our practice to close the peritoneum in order to minimize direct contact of the mesh with adjacent organs, mainly the bowel. This technique aims to correct both cystocele and apical prolapse. Rectocele repair is concomitantly done via the vaginal route for all patients. Mid-urethral sling is done for patients with demonstrated clinical stress urinary incontinence.

## Results

The demographic features of the patients and a summary of their pre- and post-operative data are presented in Table 1.

**Table 1:** Demographic and pre- and post-operative data of patients. POP-Q: pelvic organ prolapse quantification; RR: rectocele repair; SUI: stress urinary incontinence; TVTO: transvaginal tension-free vaginal tape obturator

Case	Age (years)	BMI (kg/m^2^)	Initial POP-Q: Aa,Ba,Ap,Bp,C	intervention	Postop POP-Q Aa,Ba,Ap,Bp,C	follow-up (months)	POP-Q post-partum: Aa,Ba,Ap,Bp,C
1	30	29	-1,+1,-1,+1,+2	SCP, TVTO, RR	-3,-3,-3,-1,-7	74	-3,-3,-3,-1,-7
2	37	27	-2,+2,-1,0,+2	SCP, TVTO, RR	-3,-1,-3,-3,-6	72	-3,-1,-3,-3,-6
3	33	26	-1,+1,-1,+1,+2	SCP, RR	-3,-3,-3,-1,-6	66	-3,-3,-3,-1,-6
4	37	29	-1,+1,-1,+1,+1	SCP, TVTO, RR	-3,-2,-3,-1,-6	56	-3,-2,-3,-1,-6
5	34	32	-1,+1,+1,+2,+1	SCP, TVTO, RR	-3,-2,-3,-2,-6	44	-2,-0,-3,-2,-6
6	28	34	-1,+1,-1,+1,+3	SCP, RR	-3,-3,-3,-2,-7	34	-3,-3,-3,-2,-7

Pregnancy and fetal outcomes are presented in Table 2.

**Table 2:** Pregnancy and fetal outcome after sacrocolpopexy. SCP: sacrocolpopexy

Case	Parity	Time till pregnancy after SCP (years)	Intrapartum findings during Cesarian section	Fetal outcome
1	5	3	Unremarkable	2.9 kg (alive)
2	5	4	Thick adhesion band on anterior uterus; blood loss of 1L	3.1 kg (alive)
3	3	5	Placenta previa, fetus with renal anomaly & anhydrominous	Preterm delivery 1.1 kg - neonatal death at 1 week of age
4	8	1	Uterus tilted to the right at 30°	3.9 kg (alive)
5	5	4	No records of cesarean section, patient reported uneventful cesarean section at another hospital	3 kg (alive)
6	2	1	Unremarkable	2.3 kg (alive)

Four cases required a concomitant transvaginal tension-free vaginal tape obturator (TVTO). Pregnancy was achieved at one to five years following surgery. The pregnancies were uneventful in all cases with good fetal outcomes, except in patient 3, who developed placenta previa and fetal hydropic features. This patient was looked after by the maternal-fetal medicine team and was planned for a preterm delivery at 31 weeks for fetal reasons (baby weight=1.1 kg). Neonatal death occurred at one week of age. Obstetricians performed all the cesarean sections with no reports of intraoperative difficulties or excessive bleeding.

All patients were delivered by cesarean section at our institute except case number five. She had her cesarean section at another institute, which the patient reported being uneventful. Postpartum follow-up for these patients to assess POP varies between two months to five years. The degree of prolapse was evaluated using POP-Q scoring system. A prolapse of stage 2 and above was considered a recurrence in any component during the post-operative assessments. Only one patient had a recurrent symptomatic vaginal bulge, and her husband reported feeling like a vaginal ring during intercourse. The vaginal assessment showed isolated stage 2 anterior compartment prolapse. There was no mesh erosion, but the tape was perceived superficial to the vaginal mucosa and was found to be under tension. For that, the patient underwent redo-cystocele repair and partial (right side) resection of the TVTO tape. Postoperatively this patient remains continent for urine with no more cystocele and improved sexual function.

During their pregnancies, none of the patients reported unusual pain that could be attributed to the tension of the mesh in the third trimester. None of the patients showed recurrent apical prolapse either post-operative or post-partum. No patients had recurrent or de novo stress urinary incontinence. Two patients got pregnant twice after SCP; one of them had a miscarriage that required evacuation and curettage, and the other one had a spontaneous complete miscarriage. All patients were asymptomatic for POP during the last interview.

## Discussion

High-grade POP is a stressful clinical condition, especially for young women seeking future pregnancy. There are minimal data to estimate the probability of recurrent prolapse after subsequent pregnancy and delivery. Published studies are limited to case reports and small case series, spanning multiple decades like the Wieslander study [6]. Most experts advise patients to avoid getting pregnant after undergoing major pelvic reconstructive surgery.

It is our practice to recommend family completion prior to having the surgery done as much as possible. The effect of pregnancy and childbirth on the recurrence of pelvic organ prolapse is not well-reported [6]. Balsak reported a case of vaginal birth after SCP with a favorable outcome and no recurrence of prolapse [7]. Few other small case series recommended cesarean section as the mode of delivery. In the lack of evidence, we decided to deliver our patients by cesarean section. Another issue that lacks evidence is how soon patients can get pregnant after SCP. We advised our patients to delay pregnancy till at least 12 months after surgery. One reason for this is to ensure adequate time for mesh integration to occur. Another reason is that this duration will give us a window of time to deal with any encountered complications of surgery prior to pregnancy.

In our study, a single surgeon consistently performed all the surgical procedures, thus eliminating confounding biases due to operative variations. Furthermore, all patients except one were delivered in the same institute; therefore, data on the antenatal and operative records and fetal outcomes were available. Very few studies have previously reported pregnancy outcomes and recurrence risk of prolapse after laparoscopic SCP [2,7-9]. Different surgical techniques have been used in each study; three studies reported using a single mesh posterior to the cervix [2,9,10], one used anterior and posterior meshes [10], and one used a technique similar to ours, i.e., a mesh wrapped around the cervix [8].

There isn't a set period of time for ideal follow-up that can help predict when prolapse may happen again. Two years is the maximum post-delivery follow-up period that has been documented in the literature with published objective evaluations [10]. In our study, we followed up with our six patients by having objective evaluations performed over 34 to 74 months by the same surgeon. The course of pregnancy in the presence of a mesh is another noteworthy issue. We were concerned about the constantly growing uterus and the chance that women who had mesh implants would have unusual pelvic pain during pregnancy, which could cause irritation and lead to premature labor. None of these worries came true.

Our results also lend weight to previously published data on pregnancy outcomes, which showed that all patients had favorable fetal outcomes and no prenatal or intraoperative problems. Patients in one published study received a Doppler examination to check for alterations, which was unaffected [8]. Five healthy infants in our study were delivered at terms consistent with their gestational age. One infant was born iatrogenic premature due to possible fetal impairment. It eventually became connected to congenital renal disease. After one week, the newborn died. Data on three patients who had complications, free legal abortions following SCP, were published by Emmanuel et al. [11]. In our investigation, two patients (cases three and four) each had two pregnancies, one of which was carried to term and the other of which ended in early miscarriage for each patient. With no difficulties, one of these two individuals needed dilatation and curettage. So, based on these few examples, we may conclude that there is no need to worry about the difficulty of obtaining dilatation and curettage in the future for miscarriage or other gynecological reasons.

We may infer from our short case series that laparoscopic SCP is still the preferred method for treating women with high-grade POP, which is consistent with the findings of other research [4, 12]. Reoperation for recurrent POP occurs seldom. POP recurrence was reported to be 4.2% following laparoscopic SCP in a cohort study by Sato et al. [13]. The outcomes were not significantly changed by a spontaneous pregnancy and subsequent cesarean procedure. Our study may be among the most thorough case series to evaluate pregnancy following SCP. Our findings indicated that pregnancy and a subsequent cesarean section had little to no impact on prolapse recurrence, and there were no negative pregnancy outcomes.

## Conclusions

For individuals with high-grade POP who want to preserve their fertility and have more children in the future, laparoscopic SCP is a viable and reliable treatment option. Apical prolapse recurrence appears to be negligible following SCP. Cesarean section is the preferred delivery method at this point in the clinical evidence. Our research will enable doctors to provide young women with high-grade POP fertility choices. To validate our findings in the future, more research with bigger sample numbers and more variety is required.
